# Recent advances in understanding synaptic abnormalities in Rett syndrome

**DOI:** 10.12688/f1000research.6987.1

**Published:** 2015-12-22

**Authors:** Michael Johnston, Mary E. Blue, Sakkubai Naidu

**Affiliations:** 1 Developmental Neuroscience Laboratory, Kennedy Krieger Institute, Johns Hopkins University School of Medicine, Baltimore, MD, USA; 2 Department of Neurology, Johns Hopkins University School of Medicine, Baltimore, MD, USA; 3 Department of Pediatrics, Johns Hopkins University School of Medicine, Baltimore, USA; 4 Department of Physical Medicine and Rehabilitation, Johns Hopkins University School of Medicine, Baltimore, MD, USA; 5 Department of Neuroscience, Johns Hopkins University School of Medicine, Baltimore, MD, USA; 6 Department of Neurogenetics, Kennedy Krieger Institute, Johns Hopkins University School of Medicine, Baltimore, MD, USA

**Keywords:** X-linked, Glutamate, Rett Syndrome

## Abstract

Rett syndrome is an extremely disabling X-linked nervous system disorder that mainly affects girls in early childhood and causes autism-like behavior, severe intellectual disability, seizures, sleep disturbances, autonomic instability, and other disorders due to mutations in the MeCP2 (methyl CpG-binding protein 2) transcription factor. The disorder targets synapses and synaptic plasticity and has been shown to disrupt the balance between glutamate excitatory synapses and GABAergic inhibitory synapses. In fact, it can be argued that Rett syndrome is primarily a disorder of synaptic plasticity and that agents that can correct this imbalance may have beneficial effects on brain development. This review briefly summarizes the link between disrupted synaptic plasticity mechanisms and Rett syndrome and early clinical trials that aim to target these abnormalities to improve the outcome for these severely disabled children.

## Introduction

Rett syndrome (RTT) is characterized by severe neurodevelopmental delay in psychomotor development that occurs predominantly in girls and is due to mutations in the gene on the X chromosome that codes for the transcription factor methyl CpG-binding protein 2 (MECP2) ^1, 2^. MECP2 is a transcription factor and pleiotropic protein that regulates the expression of numerous genes, including brain-derived neurotrophic factor (BDNF) ^3^, and can mediate transcriptional activation, mRNA splicing, and post-translational processing of microRNAs ^4, 5^. A phenotype similar to RTT has also been described in children with mutations in the CDKL5 and FOXG1 genes ^6, 7^. Girls with RTT often present with features similar to autism with little or no speech development, poor contact with others, and stereotyped movements, including characteristic repetitive hand-wringing movements ^8^. Girls with RTT typically undergo a postnatal regression phase during the first 3 years after birth in which hand wringing, seizures, loss of hand use, and loss of expressive language emerge ^9, 10^. Clinicians who have seen patients with RTT are usually able to identify others because of their unique combination of physical and neurologic features. In addition to autistic features and hand-wringing movements, the characteristic behavioral abnormalities in RTT include severely erratic breathing while awake with periods of alternating hyperventilation, cyanosis and apnea, severe seizures and autonomic instability in heart rate and blood pressure, and changes in the color of the extremities consistent with sympathetic instability ^11^. The incidence of sudden death is increased in girls with RTT ^12^. Developing synapses appear to be a special target of the disorder, and head circumference is usually normal at birth but then falls behind over the first year of life so that it falls into the microcephalic range ^9, 13– 16^. Neuropathologic examination of brain tissue from older girls with RTT who have died has revealed that neurons are typically too close together, suggesting a stunting of axonodendritic development ^17, 18^. This hypothesis is supported by studies of nasal epithelium from girls with RTT compared with controls, which showed that the neurogenesis of olfactory receptor neurons is normal but their maturation is blocked at the point of formation of synapses with the olfactory bulb ^19^.

## Role of excitatory synapse abnormalities in Rett syndrome

Early studies of patients with RTT, including some who had not been tested for mutations in the MECP2 gene, suggested an abnormality of glutamate excitatory synapses ^18^. For example, Hamberger *et al.* ^20^ and Lappalainen and Riikonen ^21^ in the 1990s reported that cerebrospinal fluid from girls with RTT had concentrations of glutamate but not other amino acids that were more than twice as high as those of children without RTT. Blue *et al.* in 1999 reported the first autoradiographic study of glutamate and GABA receptors in postmortem brain tissue from nine girls with RTT and 10 female controls of different ages ^22^. The authors reported that densities of N-methyl-D-aspartate (NMDA) were elevated in the frontal lobe cortex in girls younger than 8 years of age but were significantly lower in girls older than 8 years without RTT ^23^. Similar age-related changes in glutamate receptors were observed in the basal ganglia. Horská *et al.* ^24^ used magnetic resonance spectroscopy to demonstrate that glutamate was elevated in the cerebral cortex of younger girls (less than 8 years of age) with RTT, especially those with seizures, but reduced in older girls. These changes in levels of glutamate between younger and older girls correlate with the bi-phasic changes in the clinical condition of girls with RTT who have more clinical signs of excitatory activity, including seizures early in early childhood, but have reduced signs of excessive central nervous system activity later in childhood ^9^. More recently, Blue *et al.* demonstrated similar age-related changes in the NMDA1 subtype in the Bird model of RTT in mice ^25^. Taken together, the elevations in glutamate as well as in glutamate receptors in younger girls with RTT suggest that there is an abnormality of the normal homeostatic synaptic scaling by which post-synaptic receptors adjust upwards or downwards to maintain a stable level of neurotransmission ^26^. This abnormality in synaptic function probably contributes to deficits in excitatory synaptic plasticity and enhanced excitability observed in mouse models of RTT ^27– 30^.

The pathophysiology of elevated glutamate in the face of elevated NMDA glutamate receptors in RTT is not clear. The major regulator of glutamate levels in and outside the synapse is the activity of glial glutamate transporters, which normally are activated when glutamate is released into the synapse ^31^. Intra-synaptic levels of glutamate are thought to provide trophic influences on post-synaptic neurons, but glutamate flooding outside the synapse can activate excitotoxicity mediated by extra-synaptic NMDA receptors ^32^. Maezawa and Jin ^33^ reported that conditioned media obtained from Mecp2-null microglia from *Mecp2 ^tm1.1Bird/+^* mice ^34^ contain toxic levels of glutamate that is damaging to neural dendrites *in vitro* and this effect can be blocked by inhibiting glutaminase or glutamate receptors. Jin *et al.*, using the same model of Mecp2 deficiency, reported that abnormal function of the SNAT1 glutamine transporter in microglia is associated with NMDA receptor-mediated neurotoxicity, mitochondrial dysfunction, and decreased viability of microglia ^35^. Okabe *et al.* ^36^ reported that astroglia from Mecp2-null mice ( *Mecp2 ^tm1.1Bird/+^*) had downregulation of glutamate transporters that could increase extracellular glutamate to toxic levels. Lioy *et al.* ^37^ also reported that the restoration of wild-type MeCP2 in astrocytes of MeCP2-deficient mice (Mecp2 ^*Stop*^) restored normal dendritic morphology and increased levels of the excitatory glutamate transporter. We also found that cerebellar granule cells from Mecp2-null mice ( *Mecp2 ^tm1.1Bird/+^*) are more sensitive to cell death from hypoxia-ischemia and glutamate excitotoxicity compared with neurons from wild-type mice, suggesting that they also have intrinsic vulnerability to cell death ^38^. These findings are consistent with the above-cited evidence of abnormal homeostatic synaptic scaling, which is expected to degrade normal excitatory neurotransmission and activity-dependent neuronal plasticity needed for learning and memory in RTT.

## Abnormal diurnal regulation of sleep and brain glutamate levels in MeCP2 deficiency

Recently, the circadian sleep cycle in rodents has been associated with alterations in brain glutamate levels, with higher glutamate levels associated with wakefulness and sleep associated with a reduction in glutamate ^39^. We applied the relatively new technology that can measure 24-hour continuous electroencephalogram (EEG) markers of sleep cycles and correlated them with biosensor measurement of *in vivo* glutamate concentrations ^40^. We found that mice with a Mecp2 knockout mutation ( *Mecp2 ^tm1.1Bird/+^*) had abnormal behaviors and remarkably abnormal sleep cycles with long periods of sleeplessness associated with very high relative concentrations of brain glutamate. These Mecp2-deficient mice also had elevated baseline levels of cortical glutamate measured with a separate colorimetric method in postmortem tissue. These data suggest that periods of sleep disturbance that are common in girls with RTT could be associated with enhanced glutamate neurotoxicity.

## Role of GABA-containing interneurons in Rett syndrome

GABA (gamma-amino butyric acid) is the predominant inhibitory neurotransmitter in the brain and is used as a transmitter at approximately 15% of synapses, and there is ample evidence of its altered metabolism in the mice with Mecp2 deficiency. Chao *et al.* ^41^ reported that the selective deletion of MeCP2 in GABA interneurons (Viaat-Mecp22 ^/y^ mice) led to a reduction in the pre-synaptic release of GABA as well as autism-like stereotypies and RTT phenotypes in mice. Kang *et al.* ^42^ reported that there is a significant location-specific downregulation of synaptic GABA transporters in Mecp2 knockout mice ( *Mecp2 ^tm1.1Bird/+^*), and El-Khoury *et al.* ^43^ reported age- and region-specific reductions in function in GABAergic pathways in MeCP2-deficient mice (B6.129P2(c)-Mecp2. ^tm1–1Bird^). These observations along with those from the glutamate system indicate that there is a shift in the balance between excitation and inhibition in favor of excitation, as has been suggested to occur in other autism spectrum disorders ^8, 44^.

One of the most interesting recent observations on the role of altered development in GABA and glutamate-containing neurons in mice with MeCP2 mutations relates to the link between MeCP2 and visual cortical plasticity ^45^. Visual cortical plasticity associated with the occlusion of vision in one eye can be measured precisely in the laboratory in immature mice, and GABAergic interneurons play a critical role in this process ^46^. Durand *et al.* reported that MeCP2-null mice ( *Mecp2 ^tm1.1Bird/+^*) have normal visual function early in the postnatal period but that visual acuity regresses after postnatal day 35–40 and the cortex fell silent by postnatal day 55–60 ^47^. Remarkably, this effect could be prevented by genetic deletion of the NMDA glutamate receptor subunit NR2A. He *et al.* also reported that the genetic conditional deletion of MeCP2 in GABAergic parvalbumin-expressing neurons prevented local circuit inhibitory functions required for experience-dependent visual cortical plasticity ^48^. Krishnan *et al.* ^45^ reported that the MeCP2 regulates the timing of the critical period of plasticity in the primary visual cortex. Kron *et al.* ^49^ have also examined brain activity maps by using activation of the immediate early gene Fos and electrophysiology and found evidence of synaptic hyperexcitability in the cortical default mode network that was reduced by the administration of the NMDA channel blocking anesthetic ketamine. The results of these studies support the themes raised earlier in this discussion of RTT, namely that MeCP2 deficiency leads to excessive excitation versus inhibition and defective activity-dependent synaptic plasticity. Although ketamine can cause damage to developing neurons by excessively blocking the activity of NMDA receptors ^50^, its use in RTT is intended to reduce excessive activity of NMDA receptors found in RTT to normal levels. Both too little and too much activity at NMDA receptors can damage the developing brain ^51^.

## Potential new therapies based on advances in understanding Rett syndrome

Several clinical studies are examining potential therapies for girls with RTT on the basis of the basic science insights from experiments on mice with MeCP2 deficiency. Naidu *et al.* launched a randomized open-label study of oral dextromethorphan, a competitive NMDA antagonist, for seizures and cognitive function in girls with RTT from 2008 to 2014 (ClinicalTrials.gov: NCT00593957). Results of this study suggest a significant improvement in a secondary outcome of receptive speech by using the Mullen Scales of Early Learning over the course of’ 6 months but no change in seizures. Based on these promising preliminary data on cognition, Naidu *et al.* are carrying out a randomized double-blind study of dextromethorphan for girls with RTT which was started in January 2012 (ClinicalTrials.gov: NCT01520363) with support from the US Food and Drug Administration, and this study is still seeking subjects for enrollment. Other blockers of the NMDA receptor, including memantine and ketamine as mentioned above, are under discussion but have not been registered in ClinicalTrials.gov.

Published data are available on the administration of an active peptide of insulin-like growth factor 1 (IGF-1), which has been reported to be deficient in mice with Mecp2 deficiency ^52^. IGF-1 activates tropomyosin receptor kinase B (TrkB) receptors and stimulates downstream signaling through the PI3K, AKT, and mTOR (mechanistic target of rapamycin) signaling pathway to stimulate protein synthesis ^53^. Recent evidence also indicates that the positive effect of Mecp2 on mTOR is mediated by post-transcriptional processing of microRNAs that enhance mTOR activity so that Mecp2 depletion leads to a reduction in mTOR activity ^4^.

IGF-1 administration has been reported to improve behavioral functional recovery in Mecp2-deficient mice (Mecp2 ^1lox/+^ females ^54^) as well as spine density, synaptic amplitude, and PSD-95, a major constituent of the synaptic post-synaptic density in excitatory synapses ^55, 56^. Khwaja *et al.* ^52^ recently reported a preliminary assessment of the use of recombinant human IGF-1 (mecasermin) in girls with RTT. Some behavioral parameters, including anxiety and mood and an EEG parameter, seemed to improve, and the medication appeared to be safe and well tolerated ^52^. Recent preclinical data suggest that fingolimod, a sphingosine-1 phosphate receptor modulator that can increase levels of BDNF, can also reduce signs of disease in a mouse model of RTT ^57^. Valproic acid has also recently been reported to have a positive behavioral effect on mice with Rett mutations and phenotype ^58^.

## Conclusions

Mice with Mecp2 deficiency display a variety of aberrant behaviors and synaptic abnormalities that correlate very well with those in human RTT. Girls with RTT and mice with Mecp2 deficiency display elevations in both glutamate and NMDA glutamate receptors and this is likely to produce abnormal homeostatic synaptic scaling and probably contributes to impaired activity-dependent synaptic plasticity. RTT appears to involve an imbalance between elevated glutamate levels and reduced GABA levels, similar to models of autism spectrum disorders. Chaotic sleep in mice with Mecp2 deficiency has been shown to correlate with elevated baseline glutamate levels in the brain and very high elevations in glutamate associated with prolonged wakening. Several clinical studies are under way to attempt to normalize synaptic abnormalities in RTT, including the studies by Naidu *et al.* with dextromethorphan, a clinically approved competitive NMDA receptor blocker. A randomized un-blinded trial of dextromethorphan showed improvement in receptive language in girls with RTT, and a randomized trial of this drug is under way. Preclinical studies suggest that low-dose ketamine, a non-competitive NMDA blocker, might be useful for improving the connectivity of brain circuits affected in RTT and improving function. Human recombinant IGF-1 has also shown benefit in mice with Mecp2 deficiency, and a preliminary study in girls with RTT showed that it was well tolerated and may have some benefits.
